# 

**Published:** 1919-01-11

**Authors:** 


### King Edward's Hospital Fund for London.

A meeting of the General Council of King Edward's Hospital Fund for London was held on January 6 at the offices of the Fund, 7 Walbrook, E.C. 4. The Rt. Hon. James W. Lowther, M.P., the Speaker of the House of Commons, in the chair. The order of appointment, signed by the Governors, of the members of the General Council, members of Committees, and officers for the year 1919 was read. The resolutions providing for the work of the Fund for 1919, which were approved at the meeting of the Governors and General Council, held at St. James's Palace on December 17, 1918, were formally adopted.

### Forthcoming Events.

Gresham College, E.C.—Four lectures on "Physic" will be delivered by Sir Robert Armstrong-Jones, M.D., on Tuesday, Wednesday, Thursday, and Friday. January 14, 15, 16, and 17, at Gresham College, Basinghall Street, E.C., at 6 p.m. The subjects will be "Heredity," "Venereal Diseases," "Alcohol," and "Influenza."

### THE COLLEGE OF NURSING

### (Limited by Guarantee).

### PUBLIC MEETING.

A meeting to discuss the Bill for the State Registration of Nurses, drafted by the College of Nursing, Limited, will be held at the Royal Society of Medicine, 1 Wimpole Street, W., on Thursday, January 23. The Hon. Sir Arthur Stanley will take the chair at 8 p.m., and the speakers will be Miss Musson, R.R.C., Matron, General Hospital, Birmingham, Professor Glaister, Glasgow, and Sir Cooper Perry, Honorary Secretary of the College of Nursing. It is hoped that all members of the College in London will be present and bring a friend or friends. The meeting is open to all those interested in the State registration of nurses. Copies of the Bill drafted by the College of Nursing may be procured from the Secretary of the College, No. 6 Vere Street, London, W. 1, or from Messrs. Eyre and Spottiswoode, Ltd., East Harding Street, E.C. 4, at 3d. each, or 4d. post free.

### EDINBURGH CENTRE.

The last lecture of the course arranged in conjunction with the National Council for Combating Venereal Diseases was given on Tuesday, December 17, in the City Chambers, Edinburgh, by Mrs. Blaikie, M.D., on "Marriage and Parenthood." Miss White, General Superintendent of the Q.V.J.N.I. for Scotland, presided.

This course of lectures has been very well attended and much appreciated by the nurses. Miss Gill, R.R.C., President of the Edinburgh Centre of the College of Nursing, is arranging for a further series of lectures on other subjects to be given in the early part of the year. A syllabus will be issued later.

### ABERDEEN CENTRE.

(Local Representative: Miss E. Edmondson, R.R.C., Royal Infirmary, Aberdeen.)

The following course of Post-Graduate Lectures will be held at the Aberdeen Royal Infirmary at 8 p.m. on the last Thursday of each month from January to May 1919: January 30, 1919: Asepsis in Surgical Wards. February 27: Some Points in Treatment of Ophthalmic Cases. March 27: Hygiene in Relation to Nursing. April 24. Venereal Diseases. May 29: The Relation of Gynæcology to Obstetrics.

### Matrons' and Sisters' Appointments.

Evelina Hospital for Children, Southwark.—Miss Cicely Allen has been appointed night sister. She was trained at King's College Hospital.

Baptist College Hospital for Pensioners, Manchester.—Miss Esther Wolstenholme has been appointed matron. She was trained at Monsall Fever Hospital and at Salford Royal Hospital, Manchester. She has since done private nursing attached to Preston Royal Infirmary, and has undertaken day and night sister's duty at the Monsall Hospital, at Queen Mary's Hospital, Stratford, Longford Hall Hospital, Manchester, and Nell Lane Military Hospital, West Didsbury, Manchester.

Ladywell Sanatorium, Salford.—Miss Ellen H. Wilding has been appointed home sister and deputy-matron. She was trained at Blackburn Infirmary, where she was later charge nurse of the children's wards. She has been ward sister of the diphtheria block at Ladywell Sanatorium, and night sister at the Children's Hospital, Leasowe, Cheshire. Miss Ethel M. Wilson has been appointed sister. She was trained at the Sheffield Union Infirmary, later being sister there; has been staff nurse at the South-Eastern Fever Hospital, London; and has held posts at the Norwich Fever Hospital, the Hospital for Infectious Diseases, Lodge Moor, Sheffield, and the Monsall Fever Hospital, Manchester.

Royal Albert Edward Infirmary and Dispensary, Wigan.—Miss Eva Jackson has been appointed night sister. She was trained at the Norfolk and Norwich Hospital, Norwich, where she has since been sister.

Royal Victoria and West Hants Hospital, Lowther Road Branch, Bournemouth.—Miss A. E. Ellis has been appointed matron. She was trained at the East Sussex Hospital, Hastings; has been ward sister at Lyme Regis Cottage Hospital, and matron of Axminster Cottage Hospital.

The Infirmary, Beckett Street, Leeds.—Miss Effie Brown has been appointed second assistant matron and home sister. She was trained at the St. Pancras Infirmary, London, and has been night superintendent, theatre sister, and ward sister at St. Luke's Hospital, Halifax. Miss Brown holds the certificate of the Central Midwives Board.

